# The Videofluorographic Swallowing Study in Rheumatologic Diseases: A Comprehensive Review

**DOI:** 10.1155/2017/7659273

**Published:** 2017-06-15

**Authors:** Ambra Di Piazza, Federica Vernuccio, Massimo Costanzo, Laura Scopelliti, Dario Picone, Federico Midiri, Francesco Salvaggi, Francesco Cupido, Massimo Galia, Sergio Salerno, Antonio Lo Casto, Massimo Midiri, Giuseppe Lo Re, Roberto Lagalla

**Affiliations:** ^1^Section of Radiology-Di.Bi.Med., University of Palermo, Palermo, Italy; ^2^Unit of Colorectal Surgery, Department of Medical, Surgical, Neurological, Metabolic and Ageing Sciences, Second University of Naples, Naples, Italy; ^3^Department of Surgical, Oncologic and Stomatologic Diseases, University of Palermo, Palermo, Italy

## Abstract

Autoimmune connective tissue diseases are a heterogeneous group of pathologies that affect about 10% of world population with chronic evolution in 20%–80%. Inflammation in autoimmune diseases may lead to serious damage to other organs including the gastrointestinal tract. Gastrointestinal tract involvement in these patients may also due to both a direct action of antibodies against organs and pharmacological therapies. Dysphagia is one of the most important symptom, and it is caused by failure of the swallowing function and may lead to aspiration pneumonia, malnutrition, dehydration, weight loss, and airway obstruction. The videofluorographic swallowing study is a key diagnostic tool in the detection of swallowing disorders, allowing to make an early diagnosis and to reduce the risk of gastrointestinal and pulmonary complications. This technique helps to identify both functional and structural anomalies of the anatomic chain involved in swallowing function. The aim of this review is to systematically analyze the basis of the pathological involvement of the swallowing function for each rheumatological disease and to show the main features of the videofluorographic study that may be encountered in these patients.

## 1. Introduction

Autoimmune connective tissue diseases are a heterogeneous group of pathologies that affect about 10% of world population with chronic evolution in 20% up to 80% [1, 2]. Rheumatic diseases, arthritis and other diseases of muscles, joints, and bones, are common and have an important impact on the health and well-being of nearly 50 million Americans [1, 2]. Children maybe also be affected: it has been reported that nearly 300,000 American children suffer from rheumatic diseases and the most common is juvenile idiopathic arthritis [2].

Inflammation and damage to joints may also lead to serious damage to other organs, being responsible of coexisting disease and disability. For this reason, rheumatic diseases are a more frequent cause of activity limitation than heart disease, cancer, or diabetes [3].

The latest figures regarding rheumatic diseases show that they led to $127.8 billion in medical costs in the U.S [2], more than the $124.6 billion in costs for cancer care [4]. During the early stages of rheumatic disease, most of the costs are related to direct medical expenses for aggressive treatment regimens. Thereafter, the cost profile shifts with the incorporation of indirect costs related to work limitations. Reducing indirect costs, such as work limitations or even loss of work, can save the U.S. more than $47 billion per year [5].

As described before, rheumatic diseases can cause damage to vital organs, including the lungs, heart, nervous system, kidneys, skin, and eyes [6], and the majority of patients affected develops gastrointestinal tract involvement that may be caused by a direct action of antibodies against organs but also depends on pharmacological therapies [7].

In order to prevent life-threatening complications, early diagnosis and treatment of dysphagia is very important, considering also that it can lead to pneumonia, malnutrition, dehydration, and increased mortality [8].

There are over 30 autoimmune rheumatic diseases. Some of the most common are rheumatoid arthritis (RA), systemic lupus erythematosus (SLE), gout, systemic scleroderma (SSc), inflammatory myopathies (IM), juvenile idiopathic arthritis (JDM), Sjogren's syndrome (SS), sarcoidosis, spondylarthritides, and polymyalgia rheumatic and systemic vasculitis. Among them, esophageal function is more often impaired in SSc, IM, sarcoidosis, and SS [9, 10].

Moreover, orofacial manifestations occur frequently in rheumatic diseases and usually represent the early signs of disease or of its activity that are still neglected in clinical practice.

## 2. Anatomy and Physiology of Swallowing

Swallowing is a complex function enabling forwarding of food and saliva from the mouth into the stomach. Eating and swallowing in particular are complex behaviors that involve both volitional and reflexive mechanism of different nerves and muscles. During deglutition, it is important not just a correct bolus transit from oral cavity to the esophagus but also to manage airway protection. We may recognize three different phases of swallowing process: the oral, pharyngeal, and esophageal ones. Oral phase is also divided into preparatory and propulsive stages.

After liquid assumption into the mouth, the bolus is firstly held on the tongue surface against the hard palate with the upper dental arch or in the anterior part of the mouth floor. In order to prevent the liquid bolus leaking into the oropharynx before the swallow, the oral cavity is excluded posteriorly by the soft palate and the tongue contact.

Propulsive stage is dominated by tongue movements that permit a reduction in size and to soften food through mastication and salivation and allow the transit of the bolus into the pharynx.

Pharyngeal phase is characterized by two crucial biological features: food passage through the pharynx and the upper esophageal sphincter (UES) to the esophagus and airway protection, excluding larynx and trachea from the pharynx.

After that, the bolus transits to the esophagus, a tubular structure, that begins with the lower part of the UES and ends with lower esophageal sphincter (LES), which is also tensioned to prevent regurgitation from the stomach. The first part of the organ (cervical esophagus) is mainly composed by striated muscle, while the thoracic esophagus (second and third part) is composed by smooth muscle. Thus, bolus transit starts with voluntary movements that allow the passage through UES; on the other hand, the transit in the thoracic esophagus is regulated by autonomic nervous system. The peristalsis wave involved in the last two-third of the esophagus is composed by two main parts: the first is characterized by relaxation and the second by contraction to propel the bolus. Also, gravity has a role in the propulsion phase [11].

## 3. VFSS

The videofluorographic swallowing study (VFSS) or modified barium swallowing study [12] is a useful radiologic procedure to detect swallowing disorders, allowing to make an early diagnosis and to reduce the risk of gastrointestinal and pulmonary complications [13, 14].

VFSS helps to identify not only functional but also structural anomalies of anatomic regions studied during the procedure [15].

Sequential videoradiographic images are captured during all the phases of swallowing [12].

Awareness and cooperation by the patient are essential in order to perform a diagnostic procedure and having something by mouth, and smoking or chewing should be forbidden for many hours prior to the VFSS [15]. After positioning the patient in the lateral view, the VFFS starts and evaluates pharyngoesophageal segment, lips, tongue, nasal cavity, and cervical rachis [16].

Lateral view is essential to study critical valves opening and closure and pressure modifications which oropharyngeal and cervical esophagus are subjected to during swallowing. The anterior-posterior view is fundamental to assess structural and functional asymmetry [12].

Swallowing abnormalities do not always occur during the first swallows, so it is generally necessary to repeat several swallowing in order to make anomalies manifest.

The radiologist has to focus on bolus' timing, flowing and clearance, and the airway invasion, detecting when aspiration and/or penetration occurs [17].

During aspiration, ingested material goes into the trachea below the level of the true vocal folds, while during penetration, bolus material moves into the laryngeal vestibule, down to the level of the vocal folds, without crossing the vocal folds [18].

The medical report of VFSS (Table 1) should contain information related to the patient anamnesis and disease and those desumed during the procedure [17].

## 4. Swallowing Disorders

Dysphagia could be defined as the difficulty or failure of this mechanism and is often associated with impaired swallowing function. It is a condition in which disruption of the swallowing process interferes with a patient's ability to eat but also can result in aspiration pneumonia, malnutrition, dehydration, weight loss, and airway obstruction. For this reason, compromised swallowing may influence also safety, efficiency, or adequacy of nutritional intake.

Swallowing disorders can occur at any stage in the swallowing process, which are, as described before: oral phase, pharyngeal phase, and esophageal phase. The first is characterized by chewing and moving food or liquid into the throat, the second consists in starting the swallowing reflex, squeezing food down the throat, and closing off the airway to prevent food or liquid from entering the airway (aspiration) or to prevent choking, and the third results in relaxing and tightening the openings at the top and bottom of the feeding tube in the throat (esophagus) and squeezing food through the esophagus into the stomach.

Signs and symptoms of oral or pharyngeal dysphagia include coughing or choking with swallowing, difficulty initiating swallowing, food sticking in the throat, bringing food back up, sometimes through the nose, sialorrhea, unexplained weight loss, change in dietary habits, nasal regurgitation, change in voice or speech (wet voice) [19, 20], and recurrent pneumonia.

Talking about esophageal dysphagia signs and symptoms includes sensation of food sticking in the chest or throat, change in dietary habits, recurrent pneumonia, heartburn, belching, sour regurgitation, and water brash [21, 22].

In this review, we systematically described typical features of swallowing disorders in rheumatic pathologies mainly evaluating functional disorders and correlating pathological features and videofluorographic findings.

VFSS findings provide information about disease severity through the evaluation of swallowing deficits and could have also a role in the therapy management of the patient.

## 5. Scleroderma

SSc is a multisystemic chronic disease characterized by abnormalities of small blood vessels (vasculitis) and extent fibrosis of both the skin and internal organs [7]. Due to vasculitis and fibrosis, a direct damage to gastrointestinal organs such as vascular injury and consequent ischemia, neurodegeneration, muscular atrophy, and fibrosis could be induced [7, 23].

In 1994, Sjogren proposed a progression of SSc characterized by gastrointestinal involvement, vascular damage, neurogenic impairment, and myogenic dysfunction with replacement of normal smooth muscle by collagen fibrosis and atrophy. For these patients, gastrointestinal involvement represents the second most common site of damage caused by the pathology, both in limited cutaneous SSC and in diffuse cutaneous SSC, affecting more than 80% of patients. In particular, when the replacement of the smooth muscle layers of the esophagus by fibrous tissue takes over, muscle contraction diminishes and the motility could be modified.

It has been reported that 87% of patients with progressive systemic sclerosis complained from dysphagia [24].

Other mechanisms may affect swallowing function, such, for example, perioral skin and temporomandibular joint limitation may lead to difficulty of the oral phase of deglutition or mucous membrane atrophy may lead to impaired taste, eating problems, and, consequently, weight loss [24].

Esophageal involvement may occur with symptoms of heartburn and dysphagia [25].

However, symptoms may be poorly correlated with abnormalities [26, 27].

Poor emptying of the esophagus, immunosuppressive therapy, and acid suppression are also predisposing factors to *Candida* infection [28, 29].

Consequences of the direct involvement of esophagus by the pathology are stricture formation, Barrett's metaplasia, and carcinoma [30].

More rare complications are described in literature such as esophageal left atrial fistula in CREST syndrome (calcinosis, Raynaud's phenomenon, esophageal dysmotility, sclerodactilia, and teleangiectasia), secondary to perforation of an ulcer in Barrett's esophagus [31].

### 5.1. VFSS Findings

VFSS in SSc is important because it investigates both oropharyngeal and esophageal swallow function evaluating both abnormalities of oropharyngeal and esophageal swallowing and the impaired clearance of the esophagus [32].

**Figure 1:**
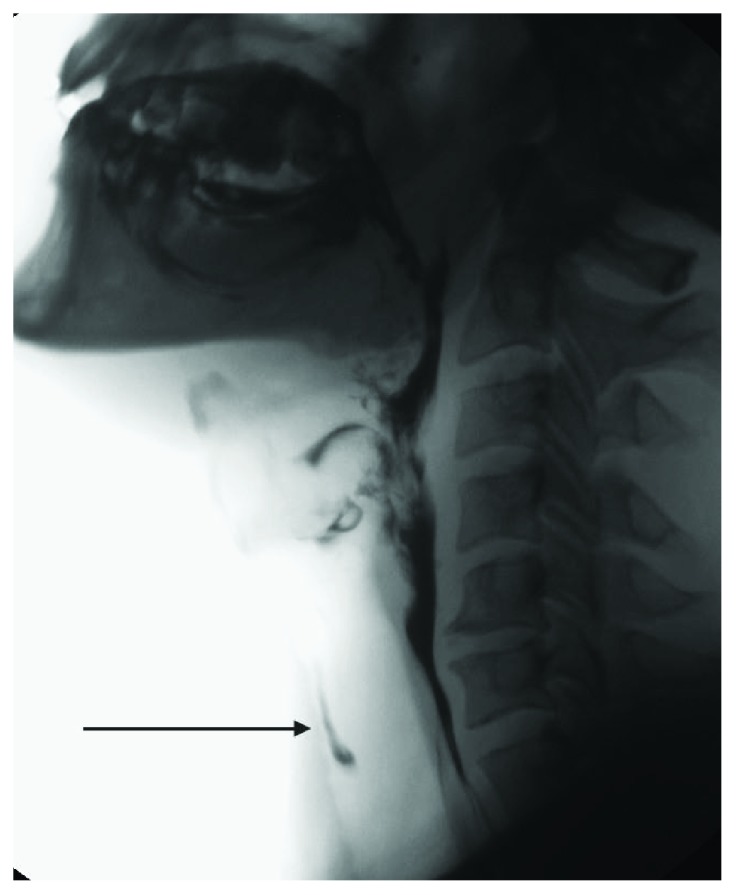
Videofluorographic study performed in a 47-year-old male with diagnosis of scleroderma. The lateral view shows the presence of intraswallowing laryngeal penetration with tracheal painting (arrow).

**Figure 2:**
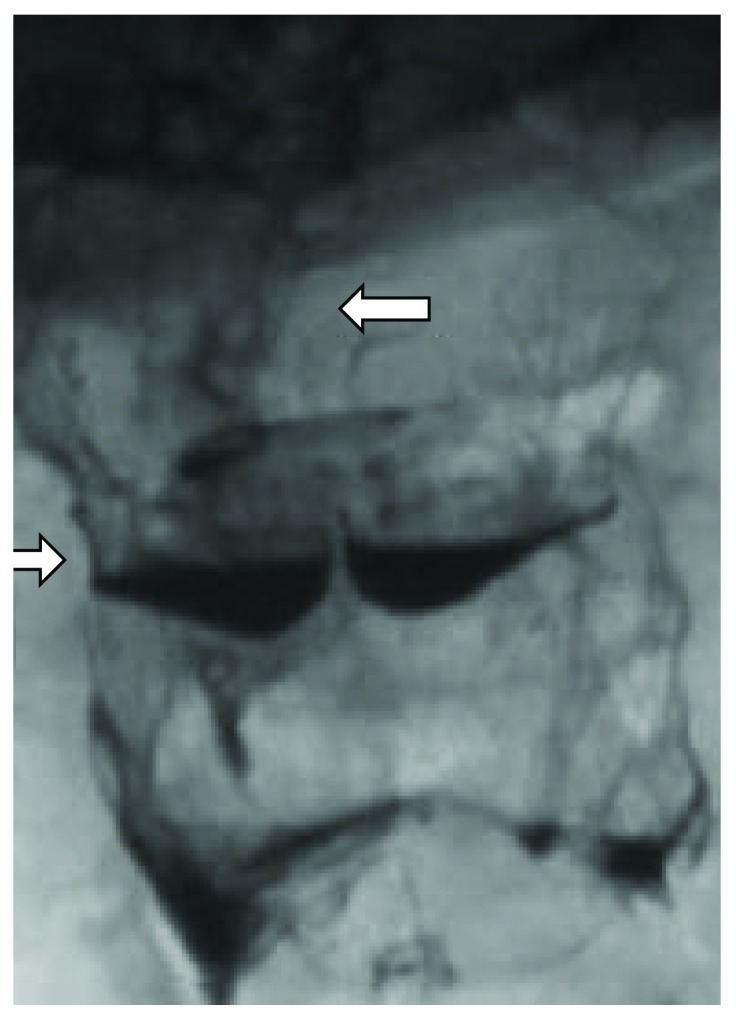
A 57-year-old female patient with scleroderma. Anteroposterior view highlights the presence of pooling contrast agent in the valleculae and pyriform sinuses due to altered motility.

VFSS in SSc may identify epiglottal tilting with associated intraswallowing laryngeal penetration (Figure 1) and pooling of contrast agent in the valleculae (Figure 2) and/or pyriform sinuses [33]. As nonspecific sign that is sometimes detected is the presence of hypertrophy of lingual and/or palatine tonsils.

**Figure 3:**
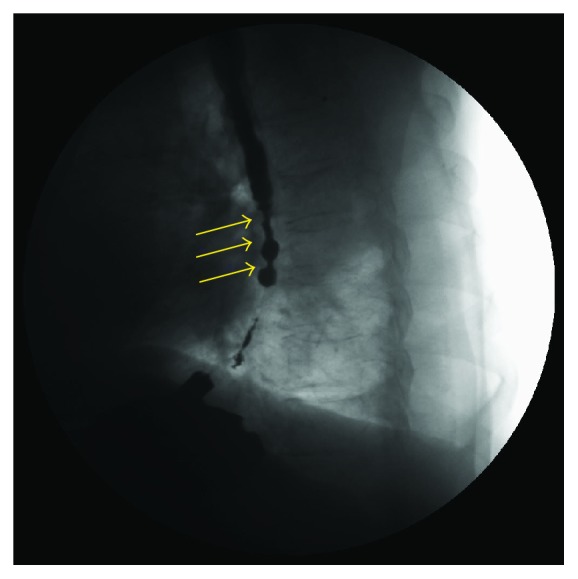
A 45-year-old female with scleroderma. Exam performed in the supine position, in the anteroposterior view. During deglutition, it is possible to highlight the presence of multiple tertiary antiperistaltic waves (arrows) in the whole esophagus; this pattern is known as corkscrew esophagus.

During the esophageal phase study, common findings are weak or absent distal esophageal peristalsis and hypotensive LES pressure, also defined as scleroderma esophagus (even when SSc is not present). Hence, esophageal dyskinesia, esophageal dilatation, esophageal clearing deficit, hiatus hernia, and gastroesophageal reflux may be found in SSc [33]. In some cases, it is has been reported the presence of multiple antiperistaltic waves of contraction, which produced a corkscrew (Figure 3) esophagus [33].

As a consequence of esophageal dilatation sometimes associated with hiatal hernia, reflux esophagitis may be encountered: on VFSS, it will appear as a reticular mucosal pattern, particularly located adjacent to a stricture [34, 35].

Dysmotility is also responsible of impaired acid clearance with a prolongation of esophageal exposure time to gastric acid leading to gastroesophageal reflux disease (GERD), which could be considered the cause of alterations of epiglottal tilting with laryngeal penetration of contrast agent [33].

As extreme consequence of the disease, esophagus may appear with a “rubber-hose” morphology due to extent atony resulting in esophageal dilatation, with multiple waves of superficial and nonpropulsive contraction involving the entire organ. In this case, the esophageal clearing is obtained only through the upright position [33].

Concluding, severe esophageal impairment, linked to reduction or absence of peristaltic waves, reduced pressure of the lower esophageal sphincter, hiatus hernia, and delayed gastric emptying and GERD correlate with a reduction of clearing in association with changes of pressure in the LES [36, 37]. These factors may lead to inflammation and Barrett's esophagus [38].

## 6. Eosinophilic Fasciitis

Eosinophilic fasciitis, also called Shulman syndrome, is a very rare, localized fibrosing disorder of the fascia. Approximately 300 cases [39] have been reported in the medical literature. The etiology and pathophysiology are unclear [39]. It has been also classified by some authors [39] as scleroderma-like syndromes.

Eosinophilic fasciitis affects both sexes. Some reports [39, 40] suggest that women are affected with greater frequency than men. The disorder can occur at any age, but most often occurs in individuals between 30–60 years. It occurs with greater frequency in Caucasians.

The first symptoms were noticed at an average of 8.8 ± 6.1 months before diagnosis [39].

In 1974, Shulman provided an early description of eosinophilic fasciitis as a disorder characterized by peripheral eosinophilia and fasciitis that could be differentiated from scleroderma by the distinctive pattern of skin involvement that spares the digits, involves fascia rather than dermis, and is not accompanied by Raynaud phenomenon [40–43].

The internal organs (viscera) may be affected in some cases, although only mildly.

### 6.1. VFSS Findings

Considering that Eosinophilic fasciitis is a scleroderma-like syndrome, VFSS findings are similar to the ones described for SSc. In particular, it has been reported the direct involvement of gastrointestinal system, especially of the esophagus. Common features are related to the deposition of immunoglobulin complex that induces a dysmotility disorder characterized by an impairment of waves of contraction. This feature may involve the entire esophagus especially the second part and third part of the lower esophagus. An increasing involvement may induce to progression of symptoms and imaging features due to hypokinesia of the whole esophagus, which may appear atonic on VFSS, sometimes with evidence of hiatal hernia.

The incontinence of LES produce a continuous exposure to gastric acid fluids that evolve in reflux esophagitis. In advanced disease, a common finding is represented by extent atony with typical GERD findings [43].

## 7. Sjogren Syndrome

**Figure 4:**
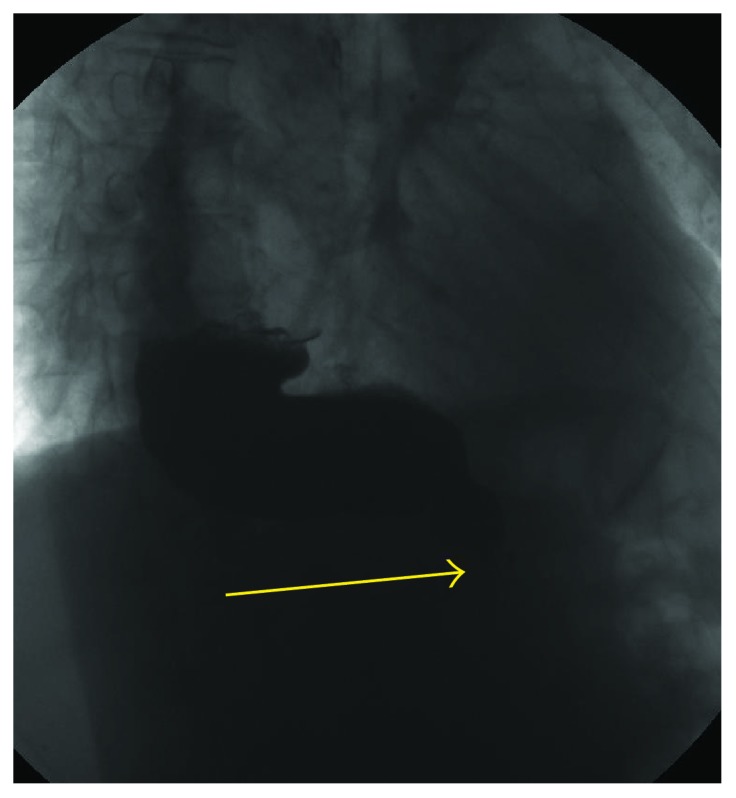
A 54-year-old woman with Sjogren syndrome. Videofluorographic swallowing study demonstrates the bird-beak (arrow) appearance of the lower esophagus, dilatation of the esophagus, and stasis of barium in the esophagus.

SS is an autoimmune disease that primarily affects the exocrine glands (mainly the salivary and lacrimal glands) and results in the severe dryness of mucosal surfaces, principally in the mouth and eyes. Symptoms can include dry skin, a chronic cough, vaginal dryness, numbness in the arms and legs, and general symptoms as fatigue, muscle and joint pains, and thyroid problems [44]. About 70% of patients affected by SS develops dysphagia [45, 46], and the causes are related to a combination of lack of saliva, esophageal dysmotility, esophageal web, achalasia (Figure 4), exocrine gland involvement, low grade myositis, and parasympathetic function damage [47–49]. Both prolonged pharyngeal transit time and absence of saliva predispose to dental caries and *Candida*. Moreover, a delayed clearance of the esophagus may lead a major exposure to acid [47, 48]. These features result in direct involvement of both pharyngeal and esophageal phases of deglutition. Moreover, esophageal symptoms sometimes do not correlate with results from investigations.

The disease predominantly affects middle-aged women, but can also be observed in children, men, and the elderly [44].

It is sometimes linked to other diseases such as rheumatoid arthritis and lupus. In Sjogren's syndrome, glands that make tears and saliva are affected causing dry mouth and dry eyes, also called as syndrome sicca [44].

### 7.1. VFSS Findings

In SS, pharyngeal transit time increases and patient needs more time to start deglutition with a resulting hesitation and in some nonpropulsive movements of tongue. It could be defined as the difficulty initiating swallowing.

**Figure 5:**
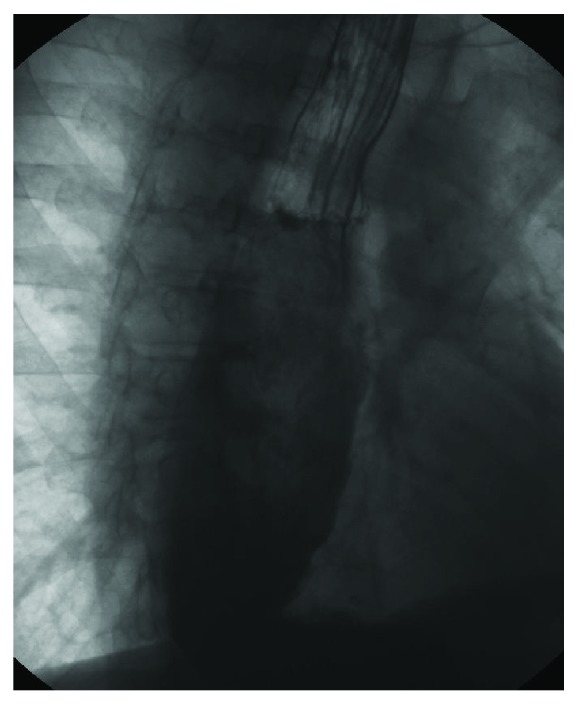
A 54-year-old woman with Sjogren syndrome. Videofluorographic swallowing study demonstrates atonic esophagus with “rubber-hose” appearance and associated achalasia.

It has also been often described aperistalsis in the upper 10 cm; in other cases, it has been reported aperistalsis in the whole esophagus especially during dry swallows (Figure 5).

The upper esophagus may be involved also by the presence of triphasic tertiary contractions or nonperistaltic contractions; however, these findings could be common also in the whole organ. In only one study, it has not been reported any abnormality of the upper or lower esophageal sphincter [50].

A shorter peristaltic contraction time of the whole esophagus in association with a faster peristaltic velocity preferably in the distal part of the esophagus has also been reported [51].

Achalasia may also be detected [51]. In this case, common findings are represented by reduction of esophageal clearing time in association with aperistalsis; moreover, especially in advanced stage of pathology, it could be recognizable an air-fluid level, classified on the base of the location in distal, intermediate, or proximal; at least, esophagus-gastric junction has been described very thin with the typical sign of mouse tail [52].

## 8. Sarcoidosis

The incidence of sarcoidosis averages 1 : 10.000 in the western world [53].

Sarcoidosis is an inflammatory, granulomatous, multisystem disorder of unclear etiology [1]. The lungs are predominantly involved, but it can entail involvement of any other organ or organ systems such as the skin, lymphatics, heart, musculoskeletal, neurological, and gastrointestinal system [54–56].

It has the highest incidence in the United States and Sweden [57]. In the United States, it is more common in African Americans with an age adjusted annual incidence rate of 35.5 in 100,000, whereas in Caucasians it is 10.9 in 100,000 [57]. The lifetime risk of developing sarcoidosis is 2.4% in African Americans compared to 0.85% in whites [58].

Sarcoidosis tends to affect individuals aged 40 years or younger [57].

While gastrointestinal involvement of sarcoidosis is seen very infrequently, esophageal involvement of sarcoidosis is extremely a rare occurrence in sarcoidosis. A review of the literature revealed only 23 published cases of esophageal involvement in sarcoidosis to date [59–62].

In the cases reported in literature, patients swallowing difficulty was defined by stenosis of the distal esophagus.

Even though, dysphagia is the most common symptom in patients affected by sarcoidosis with esophageal involvement [63]. The esophageal involvement in sarcoidosis has been classified in literature based upon two criteria: the level of involvement and the layer of involvement [62].

Focusing on the site of involvement, symptoms and diagnostic features depend on them. Superficial involvement of the mucosa may manifest macroscopically as mucosal hyperemia, discrete plaque-like or nodular lesions, with, sometimes, the appearance of Barrett's esophagitis [64]. Myopathic involvement, however, is induced by direct infiltration of skeletal muscle of the esophagus and pharynx and could be classified into three distinct classes: nodular lesions, acute myositis, and chronic myopathy [65].

Also, direct involvement of the enteric nervous plexus can cause dysphagia, and, in this case, the clinical features can mimic achalasia [66]. Development of strictures secondary to sarcoid involvement of the esophagus have also been described [67]. It has also been reported a case of worsening dysphagia resulting in both extrinsic compression by enlarge mediastinal nodes and neuromuscolar dysfunction due to direct infiltration [63, 68]. However, the lower esophagus was the most commonly involved than the upper esophagus [69].

### 8.1. VFSS

As described before, different mechanisms may produce a direct or indirect involvement of the esophagus in sarcoidosis. Videofluorography can provide information about pharyngeal phase of swallowing, status of the esophageal sphincters, and peristalsis.

Concerning the first part of the esophagus, it has been reported a significant narrowing at the level of pharyngoesophageal junction with hypertony of UES, which could be related to the direct infiltration of skeletal muscle of the esophagus and pharynx [70].

An important feature is represented by the lack of peristalsis in the esophageal body and an incomplete opening of LES after swallowing, also resulting from infiltration of both skeletal and smooth muscles.

It has also been reported a case of achalasia-like dysmotility in which VFSS findings were a mildly dilated esophageal body with barium hold-up in the distal esophagus and a bird-beak appearance of the esophagogastric junction. Pathological mechanism is due to direct infiltration of nerves and musculature of the esophageal wall [71].

In case of extrinsic compression by enlarged lymphonodes, videofluorography shows a large mass deforming the middle esophagus resulting in esophageal strictures [72].

Esophagus involvement is also been reported as a midesophageal traction diverticulum secondary to inflamed mediastinal lymphonodes [73].

**Figure 6:**
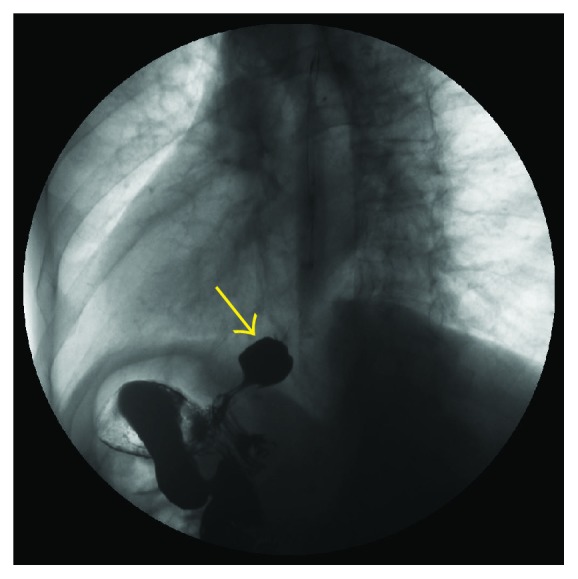
A 56-year-old man with sarcoidosis. Videofluorographic swallowing study performed in the supine position. In the lateral view, the presence of a little sliding hiatal hernia (arrow) is noticed that could not be seen in orthostatism (not shown in this figure).

As already said, there have also been cases of Barrett's esophagitis [74]. Classic radiologic features of Barrett's esophagus consist of a high esophageal stricture or ulcer, often associated with a hiatal hernia (Figure 6()) or gastroesophageal reflux. The strictures may appear as ring-like constrictions or, less commonly, as smooth, tapered areas of narrowing in the midesophagus. Barrett's ulcers typically appear as relatively deep ulcer craters within the columnar mucosa at a considerable distance from the gastroesophageal junction [75].

Occasionally, however, a reticular or villous pattern of the mucosa may be observed as the only morphologic abnormality in Barrett's esophagus [76].

## 9. Systemic Lupus Erythematosus

SLE is a chronic inflammatory disease characterized by different manifestations and following a relapsing and remitting course. More than 90% of cases of SLE occur in women, frequently starting at childbearing age [77].

Annual incidence of SLE from the 1970s to 2000s has ranged from approximately 1 to 10 per 100,000 population, while the prevalence of SLE has been estimated to range from approximately 5.8 to 130 per 100,000 population [77].

Although the specific cause of SLE is unknown, multiple genetic predispositions and gene-environment interactions have been identified [78, 79].

Gastrointestinal manifestations are common in patients with SLE. In 1985, William Osler emphasized that gastrointestinal manifestations in SLE may mimic any kind of abdominal condition [80]. In particular, dysphagia occurs in about 13% and heartburn in up to 50% of patients with SLE. Esophagitis with ulceration has been observed in 3–5% of patients [80].

The aetiopathological process causing esophageal dysmotility in patients with SLE is uncertain, but both inflammation of the esophageal muscles and vasculitic damage to the Auerbach plexus could be responsible [81].

### 9.1. VFSS

VFSS in SLE may show hypoperistalsis and aperistalsis in about 72% of patients, while abnormality low or absent contractions are found in the upper one-third of the esophagus [81].

It has been reported also a prolonged pharyngeal transit times, with no difference compared to patients with primary Sjogren's syndrome. In fact, both in SLE and in SS, the upper one-third of esophagus is mainly affected [82, 83].

The cause of the swallowing disorder is mainly related to recurrent mouth ulcers in about 30% of patients and SS in about 20%. It is also important to underline a potential cause of dysphagia represented by *Candida albicans*, especially in patients treated with immunosuppressive therapy [84]. Although involved, the LES is almost spared, if compared with other autoimmune rheumatic diseases. However, dysphagic symptoms are related to GERD due to abnormal peristalsis in both proximal and distal esophagus [29].

## 10. Idiopathic Inflammatory Myopathies

IIM is a group of systemic connective tissue disorders characterized by inflammation of the muscles used for movement (skeletal muscles), proximal symmetrical muscle weakness, decreased muscle endurance, and chronic inflammation in muscle tissue [84–86].

Idiopathic inflammatory myopathy usually appears in adults between ages 40 and 60 or in children between ages 5 and 15, though it can occur at any age [85]. The incidence of idiopathic inflammatory myopathy is approximately 2 to 8 cases per million people each year [85].

They can be subclassified into dermatomyositis, polymyositis, and inclusion body myositis (IBM) considering differences in clinical and histopathological features [85, 87].

Polymyositis (PM) and dermatomyositis (DM) involve weakness of the muscles closest to the center of the body (proximal muscles), such as the muscles of the hips and thighs, upper arms, and neck. In some cases, moreover physical difficulty, they may develop swallowing or breathing difficulty due to muscle weakness.

Symptoms are similar, but polymyositis and dermatomyositis are distinguished by a reddish or purplish rash on the eyelids, elbows, knees, or knuckles.

The GI manifestations of the idiopathic inflammatory myopathies include uncoordinated swallowing, uncoordinated esophageal peristalsis, and hiatal hernia with reflux and stricture formation [88].

From 8% to 30% of patients develops dysphagia, with higher incidence in myositis-affected patients [89, 90].

Esophageal motility is involved in every aspect considering the features of the disease: in particular, both skeletal and smooth muscle function is impaired. Especially, UES function is impaired, more than in scleroderma [91].

Involvement of striated muscles of the pharynx and upper esophagus occurs in 10–15% of cases and may lead to dysphagia and also regurgitation and aspiration pneumonia [92].

Dysphagia features depend on inflammation and dysmotility of the upper and lower esophagus and cricopharyngeal muscle dysfunction, which are responsible of a characteristic sensation of food sticking in the back of the throat or coughing with swallowing [93]. 70% of patients may present distal esophageal abnormalities in the absence of proximal esophageal involvement.

Distal dysmotility features are similar to scleroderma, but not physiopathologically related. Dysmotility of the lower esophagus is related to the duration of the primary disease [88].

Thanks to these pathological mechanisms; it is possible to recognize on one hand the formation of diverticula due to degeneration of the skeletal muscle and weakness of smooth ones, on the other hand, atony that may predispose to GERD [94].

As in other rheumatic pathologies, immunosuppressive therapy may predispose also to candidiasis and other esophagitis such as herpetic, both herpes simplex and CMV [47].

At least, it is important to remember, as in other diseases, that vasculitis may cause ulceration and even esophageal perforation; however, it is more common in children.

Patients with IBM develop dysphagia in 40–80% of cases, more than DM and PM [29].

At least, patients with dysphagia exclusively related to cricopharyngeal dysfunction have a better prognosis if they undergo myotomy [95].

### 10.1. VFSS

At a first approach, before starting the examination, dysphonia with a nasal speech could be noted. It could be also important to evaluate the involvement of diaphragm and intercostal muscles, which may cause some problems both in swallowing and in breathing during the examination [92].

The triggering of the swallowing for the voluntary phase is almost normal, but pharyngeal phase is often prolonged. UES results hypotonic in majority of cases associated with a tongue weakness and sphincter closing problem. Oropharyngeal swallowing problems include also the involvement of the one-third proximal part of the esophagus, which is hypotonic too [29].

When pharyngeal involvement is registered also in juvenile DM, it is associated with a poor prognosis, because it predisposes to a major risk of swallow dysfunction and aspiration [96].

Most common abnormalities are residual pharyngeal pooling, tongue base weakness, airway penetration, reduced UES contraction, also as a prominent, tight cricopharyngeal muscle with poor relaxation, and impaired laryngeal elevation. Aspiration during deglutition is also frequent [97].

Lower esophagus involvement is more common in distal esophagus in IBM.

VFSS features are similar to SSc. Thus, there is a peristaltic decrease that may determine expansion of the lower esophagus and a LES-impaired contraction. Moreover, dysmotility leads to GERD. When prolonged, acid exposure may cause reflux esophagitis characterized by reticular or villous pattern of the mucosa [92].

In some cases it is possible to recognize diverticula that appears, at VFSS, as round extroflession of mucosa with contrast pooling inside that may have different dimension and connection with the esophageal lumen [94].

## 11. Rheumathoid Arthritis

RA is an autoimmune disorder characterized by chronic synovial inflammation that induce joint destruction and bone erosions. Pathogenic mechanisms are not fully clear, but it is known that both genetic and environmental factors trigger an abnormal autoimmune response [98]. RA affects between 0.5 and 1% of adults in the developed world with between 5 and 50 per 100,000 people newly developing the condition each year [98].

Onset is uncommon under the age of 15, and from then on, the incidence rises with age until the age of 80 [99]. Women are affected three to five times as often as men [100].

The disease most commonly starts in women between 40 and 50 years of age [99]. A spontaneous remission may occur; however, the natural course is almost invariably persistent symptoms and a progressive deterioration of joint structures leading to deformations and disability. Potentially, any organ and tissue could be affected. Gastrointestinal involvement, especially dysphagia, is related to pharmaceutic therapies, atlantoaxial subluxation, vasculitis that leads to dysmotility, fibrosis, stricture, and ulceration. Gravity depends on disease severity or duration of RA [29, 49, 99].

### 11.1. VFSS

First part of examination may be altered by difficulty of chewing and swallowing related to direct laryngeal involvement by synovitis and nodules.

Temporomandibular joint involvement and sicca syndrome may also have a role in this phase of deglutition [101].

In the proximal esophagus, it has been reported a decreased peristaltic pressure, related to striated muscle dysfunction [102].

Characteristic features of RA are low-amplitude peristaltic waves in the lower two-third of the esophagus and impaired LES pressure, which may predispose to GERD [103].

In some cases, it could be recognized a stricture in the lower esophagus due to the presence of esophageal varices, related to Felty's syndrome that induces a nodular hyperplasia of the liver with portal hypertension [104].

In children with JRA, temporomandibular joint pain is rarely reported and it results in compromised masticatory function. Articular involvement is often associated with dysphagia.

Other findings include decrease/impairment of distal esophagus peristalsis and esophageal ulcers, maybe due to esophageal reflux [105].

## 12. Vasculitis and Complications of Antirheumatic Therapy

Vasculitis is a heterogeneous group of disease entities, whose common feature is vascular wall damage through inflammation caused by autoimmune processes. The severity of the disease depends on whether only skin and subcutaneous tissue vessels are occupied or whether organ alterations occur.

Vasculitis can also be induced by numerous drugs including nonsteroidal anti-inflammatory drugs, antibiotics, antihypertensive drugs, and so on.

Esophageal involvement in vasculitis has been largely reported in literature [106].

In this group of pathologies, it should be remembered Behcet disease, in which dysphagia is due to both oral and esophageal ulcers, but only 3–26% of patients have GI involvement [107].

In Henoch-Schonlein purpura, GI involvement includes esophageal strictures [47] and carcinoma [108].

Many drugs used to treat musculoskeletal conditions can affect swallowing in various ways.

In fact, anti-inflammatory drugs, corticosteroids, and bisphosphonates can cause esophagitis and esophageal ulceration. Moreover, gold compounds (intramuscular and oral), penicillamine, sulfasalazine, methotrexate, and other cytotoxic drugs can cause oral ulcers and other lesions to oral mucosa. Similarly, alendronate sodium has a toxic effect and a physical irritation of the mucosa caused by the pill [109].

“Pill esophagitis” in fact is characterized by retrosternal chest pain and possibly dysphagia, odynophagia, and symptoms that could be related also to GERD [30].

Candidiasis of the upper GI tract is also a frequent consequence of steroid therapy and other immunosuppressive agents. Gold-induced enterocolitis has been found histopathologically to involve the esophagus as well as the stomach and small bowel [110].

### 12.1. VFSS

Findings could be highly variable in relation to the underlying mechanisms. In fact, it could be possible to recognize a reduced UES contraction or an impaired peristalsis of two-third esophagus, from hypotony up to atony.

The presence of changes of pressure in the LES with consequent incontinence produces a continuous exposure to gastric acid fluids, evolving in reflux esophagitis up to typical GERD findings has been described [44].

Esophagitis may evolve Barrett's esophagus [74] with its classic radiologic features such as esophageal stricture or ulcer, sometimes associated with hiatal hernia or gastroesophageal reflux [75].

## 13. Conclusion

Despite the heterogeneity of the wide range of rheumatic diseases, swallowing disorders are frequently encountered in all of them.

Due to the presence of different mechanisms that may determine swallowing disorders, potentially each stage of deglutition can be altered.

The most important causes that determine a direct damage of organs involved in deglutition are fibrotic infiltration, muscle degeneration, both smooth and skeletal, vascular damage, node enlargement, which may attract part of esophagus, and joint degeneration. The role of VFSS during years, as both morphological and functional study, is already defined not only in the detection but also in the evaluation of progression of the pathology. For these reasons, it is important to consider VFSS as the gold standard in the complete functional assessment of every phase of swallowing in rheumatological diseases.

## Figures and Tables

**Table 1:** American Speech and Hearing Association (ASHA)—adult assessment template: videofluoroscopic swallowing exam form.

Proposal for VFSS template
(1) Firstly, specify patient's identificative information; past, recent, surgical, and familiar medical history; past or recent medications.
(2) Focus on the reason of the examination and patient's subjective symptoms.
(3) State the patient's position during the procedure and specify if cooperation by the patient is enough to achieve a diagnostic exam.
(4) Indicate the types of barium meal used, bolus' volumes, and textures administered to the patient.
(5) Specify if swallowing abnormalities (aspiration, penetration, swallow delay, and residue) are present or absent and if they occur before, during, or after swallow.
(6) Highlight swallowing abnormalities of every swallowing phase (oral, pharyngeal, and esophageal phase).
(7) Specify if backflow is observed during esophageal phase.
(8) Report provocative or therapeutic maneuvers.
(9) Specify and characterize the swallowing diagnosis of dysphagia or, if swallowing process is not impaired, highlight the normal limits of the different phases.
